# Linear regression analysis of Hospital Episode Statistics predicts a large increase in demand for elective hand surgery in England

**DOI:** 10.1016/j.bjps.2014.10.011

**Published:** 2015-02

**Authors:** Emily Bebbington, Dominic Furniss

**Affiliations:** a The School of Clinical Medicine, University of Oxford, The John Radcliffe Hospital, Oxford OX3 9DU, UK; b The Department of Plastic and Reconstructive Surgery, Oxford University Hospitals NHS Trust, Nuffield Orthopaedic Centre, Oxford OX3 7HE, UK; c NDORMS, University of Oxford, Botnar Research Centre, Oxford OX3 7HE, UK

**Keywords:** Dupuytren's disease, Carpal tunnel, Cubital tunnel, Trigger finger, Incidence, Workforce planning

## Abstract

**Introduction:**

We integrated two factors, demographic population shifts and changes in prevalence of disease, to predict future trends in demand for hand surgery in England, to facilitate workforce planning.

**Methods:**

We analysed Hospital Episode Statistics data for Dupuytren's disease, carpal tunnel syndrome, cubital tunnel syndrome, and trigger finger from 1998 to 2011. Using linear regression, we estimated trends in both diagnosis and surgery until 2030. We integrated this regression with age specific population data from the Office for National Statistics in order to estimate how this will contribute to a change in workload over time.

**Results:**

There has been a significant increase in both absolute numbers of diagnoses and surgery for all four conditions. Combined with future population data, we calculate that the total operative burden for these four conditions will increase from 87,582 in 2011 to 170,166 (95% confidence interval 144,517–195,353) in 2030.

**Discussion:**

The prevalence of these diseases in the ageing population, and increasing prevalence of predisposing factors such as obesity and diabetes, may account for the predicted increase in workload. The most cost effective treatments must be sought, which requires high quality clinical trials. Our methodology can be applied to other sub-specialties to help anticipate the need for future service provision.

## Introduction

Hand surgery is an important interface sub-speciality encompassing orthopaedics and plastic surgery. The importance of hand surgery is well recognised as much of the function of the upper limb and thus personal and economic productivity is dependent upon the hand's proper functioning, thus there is an overt need for a properly planned, comprehensive, hand surgery service.^2^

Financial pressures on health services worldwide make knowledge of likely future service use important for planning. It has been acknowledged that there may be some shortcomings in hand disease service provision and that effective future planning is essential to ensure high standards are maintained.^1^

Much has been made of population demographic shifts, particularly the ageing population, leading to increases in workload. However, changes in prevalence of diseases over time will also contribute to an increase or decrease in workload. It is possible that there will be a disproportionate increase in surgical procedures commonly required in elderly people, those with diabetes, and the obese, and this must be met with adequate service provision and planning.^3^ Workforce planning is integral to this, and there has been much recent interest in a supply-demand model for surgical training.^4^

In this paper we integrate two factors – demographic population shifts and changes in prevalence of disease – in order to predict future trends in demand for hand surgery in England. The Hospital Episode Statistics (HES) record all diagnoses and procedures performed within the NHS in England. We firstly analyse this HES data for four common hand surgery conditions – Dupuytren's disease, carpal tunnel syndrome, cubital tunnel syndrome, and trigger finger – from 1998 to 2011. Using linear regression, we estimate trends in both diagnosis and surgery for these conditions until the year 2030. We then integrate these estimates with age specific population data in order to estimate how this will contribute to a change in workload over time. It is hoped that this research will facilitate the planning of future service provision, and guide the allocation of funding for much needed research into optimal treatments for these conditions. Furthermore, our methodology can be applied to other sub-specialties to help anticipate the need for future service provision.

## Materials and methods

Hand disease data was collected from the Hospital Episodes Statistics (HES) website for England (www.hesonline.nhs.uk). Primary diagnosis according to ICD-10 criteria and primary intervention and operation data according to OPCS-4 were extracted for Dupuytren's disease, carpal tunnel syndrome, cubital tunnel syndrome, and trigger finger from 1998 to 2011. Data for the 0–14 age group was removed from all analyses in order to exclude congenital cases.

Corresponding operations were identified for each primary disease; for Dupuytren's disease ‘palmar fasciectomy’, ‘digital fasciectomy’, ‘revision of palmar fasciectomy’, ‘revision of digital fasciectomy’, and ‘division of palmar fascia’ (needle fasciotomy) were recorded. For the other conditions ‘carpal tunnel release’, ‘revision of carpal tunnel release’; ‘cubital tunnel release’; and ‘release of constricted tendon’ were recorded. The available data for operative procedures spanned 2000–2011.

Population data was collected from the Office of National Statistics website for England (www.statistics.gov.uk), according to age groups matching HES data (15–59, 60–74, 75+) for 1998–2011, and projected population figures for each age group in 2015, 2020, 2025, and 2030. The percentage of the population in each age group was calculated. The proportion of males in the projected population figures was compared to current figures using the paired *T*-test, in order to rule out a change in the proportions of males and females confounding results for those conditions with differing sex-specific incidences.

We undertook univariate linear regression on the diagnosis rates per person of the population for each hand disease from 1998 to 2011, and calculated 95% confidence intervals. We then extrapolated this data to 2030, calculating diagnosis rates for each age group and also as a total of all age groups.

Next, we performed univariate linear regression on the operation rates per person of the population for each hand disease from 2000 to 2011, and calculated 95% confidence intervals. We then extrapolated this data to 2030, calculating operation rates for each age group and also as a total for all age groups. The mean and 95% confidence intervals at 2015, 2020, 2025, and 2030 were recorded. Next, these results were multiplied by the predicted number of people in each population age group for 2015, 2020, 2025, and 2030 to show the predicted workload in the future.

To establish whether increased operative management, as opposed to non-operative management, of these common hand conditions could account for some increased workload, we calculated the ratio of number of operations per diagnosis for each condition. Finally, to test whether the introduction of a maximum 18 week wait from referral to treatment policy in the NHS in 2008 artificially increased the number of diagnoses and/or operations, we performed univariate linear regression of pre-and post-2008 data with analysis of covariance of the slopes.

All statistical calculations were performed GraphPad Prism version 5.00 for Windows (GraphPad Software, San Diego California USA, www.graphpad.com).

## Results

### Population statistics

**Figure 1:**
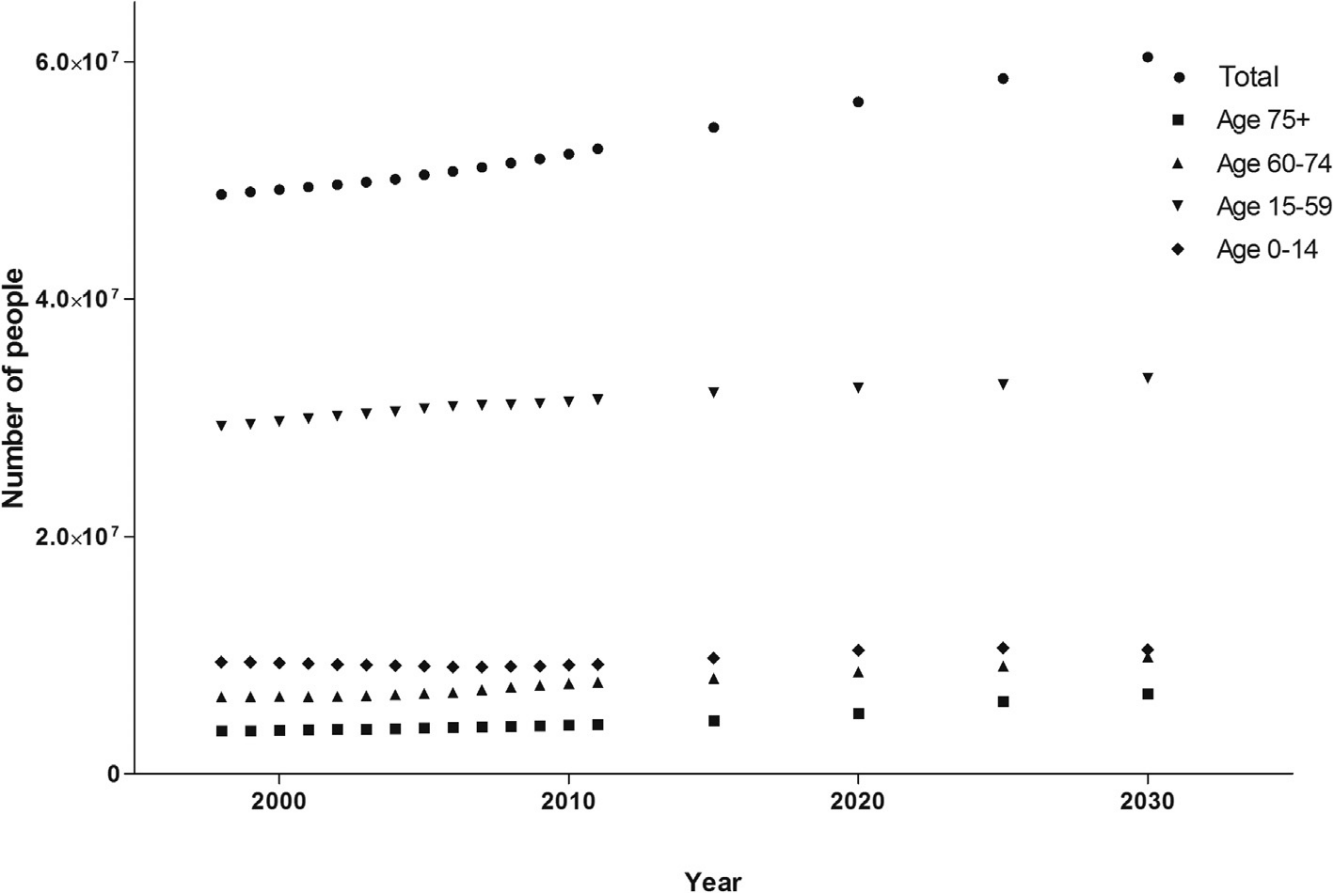
Population of England 1998–2030. Data is stratified by age groups. Source of data – the Office for National Statistics.

The population of England increased between 1998 and 2011 from 48,820,600 to 52,655,389 people, and this is predicted to continue to 2030 (60,409,534 people) (Figure 1). The proportion of the population in younger age groups (0–14 and 15–59) are predicted to decrease (2011: 17.54% and 59.83%; 2030: 17.33% and 55.19%respectively), whilst the proportion of people in older age groups (60–74 and 75+) are predicted to increase (2011: 14.68% and 7.96%; 2030: 16.30% and 11.18% respectively). The proportion of men and women is not predicted to significantly change (*p* = 0.69).

### Increasing diagnosis and surgery for Dupuytren's disease

We here describe in detail the results of our analysis of Dupuytren's Disease, presenting only summary results for the other conditions. Full data for these conditions is available in the Supplementary data.

**Figure 2:**
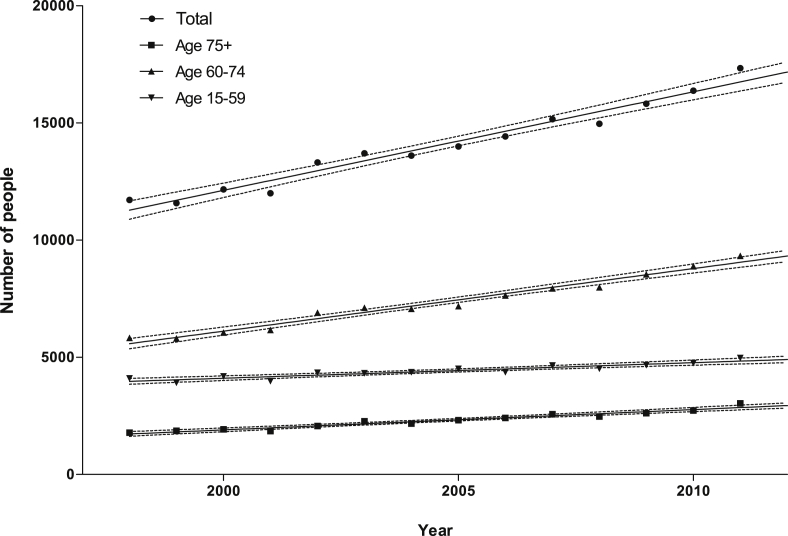
Absolute numbers of diagnoses of Dupuytren's Disease, 1998–2011. Solid lines represent the means, and dotted lines the 95% confidence intervals of linear regression analysis performed on the data. For all age groups combined *r*^2^ = 0.96.

The number of diagnoses of Dupuytren's disease is increasing; the greatest number of diagnoses is in the 60–74 age group (Figure 2). The fastest rate of growth is also in the 60–74 age group (slope mean 266.79, 95% CI 238.28–295.29). The rate of diagnosis of Dupuytren's disease per person in the population has also increased between 1998 and 2011, in the 60–74 age group and the 75 and over age group (see Supplementary data).

**Figure 3:**
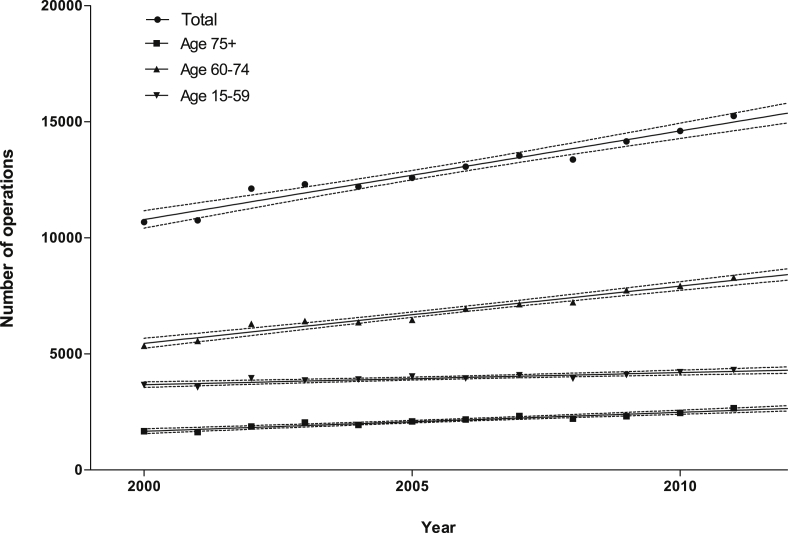
Absolute numbers of operations for Dupuytren's Disease, 2000–2011. Solid lines represent the means, and dotted lines the 95% confidence intervals of linear regression analysis performed on the data. For all age groups combined *r*^2^ = 0.96.

Current trends in operative procedures for Dupuytren's disease closely mirror the diagnoses; the 60–74 age group require the greatest number of operations for Dupuytren's disease per person of population and this is predicted to continue to 2030 (Figure 3). Again, the fastest rate of growth of operations is also seen in the 60–74 age group (slope mean 246.70, CI's 213.52–279.88).

**Figure 4:**
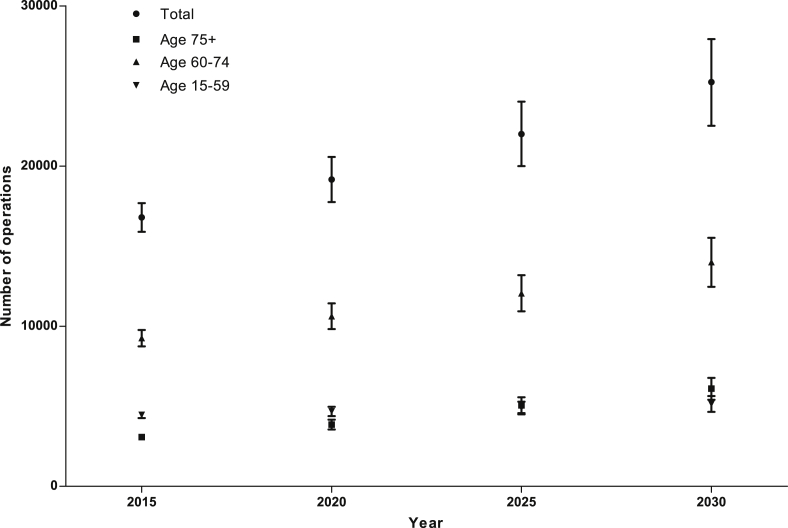
Predicted future operation rates for Dupuytren's disease. Filled symbols represent the mean and error bars the 95% confidence intervals of the estimates.

**Figure 5:**
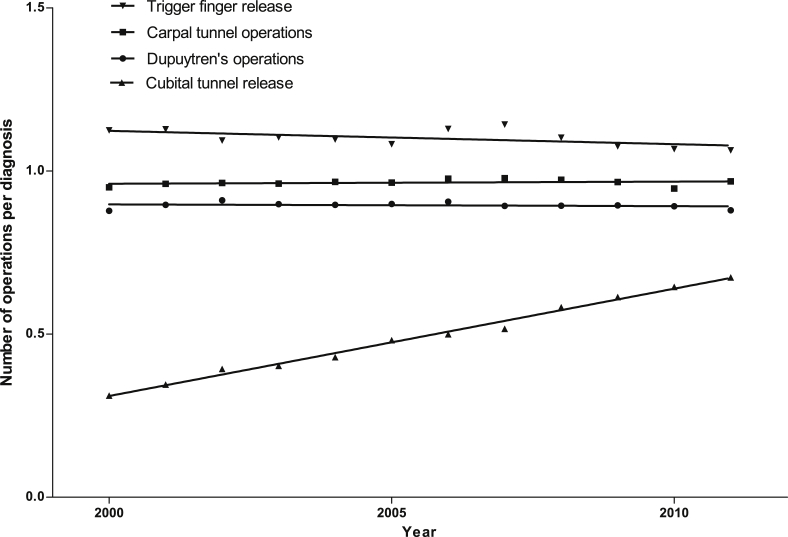
Ratio of number of operations per diagnosis for four common hand conditions, 2000 – 2011.

Future absolute operation rates for Dupuytren's disease, adjusted for the predicted population in each age group, are predicted to steadily increase over time (Figure 4, Table 1). The number of operations performed for Dupuytren's disease from 2000 to 2011 per diagnosis has remained constant at 0.89 (slope not significant, *p* = 0.51), ruling out increased operative management of Dupuytren's disease as a cause for this increase (Figure 5). The slopes of pre-2008 versus post-2008 data for Dupuytren's diagnoses were significantly different (*p* = 0.019), but the slopes for Dupuytren's operations were not significantly different (*p* = 0.15). This suggests an increased diagnosis, but not increased operative management since the introduction of the 18-week wait policy for this condition.

### Increasing diagnosis and surgery for other hand conditions

**Figure 6:**
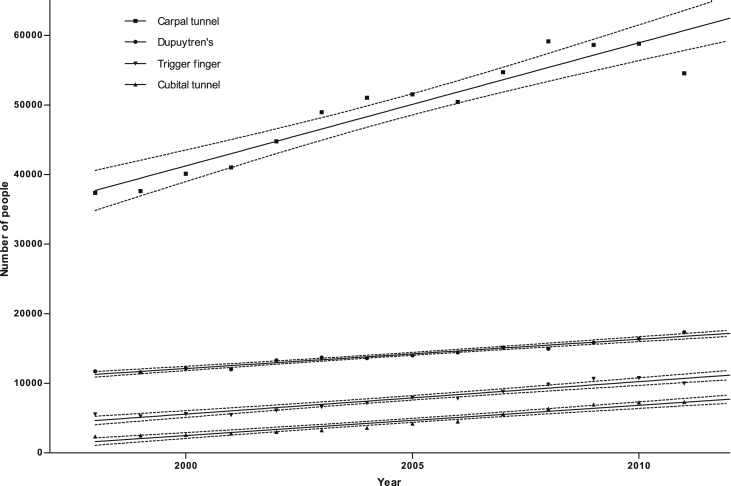
Absolute numbers of diagnoses for four common hand conditions, 2000–2011. Solid lines represent the means, and dotted lines the 95% confidence intervals of linear regression analysis performed on the data. For carpal tunnel *r*^2^ = 0.90; Dupuytren's disease *r*^2^ = 0.96; trigger finger *r*^2^ = 0.93; cubital tunnel *r*^2^ = 0.94.

In parallel with the data reported for Dupuytren's disease, the number of diagnoses for all hand diseases analysed have increased between 1998 and 2011 (Figure 6). The fastest rate of increase is in carpal tunnel disease (slope mean 1768, 95% CI 1392–2144). Furthermore, the number of diagnoses as a percentage of the population has also increased for all four conditions.

**Figure 7:**
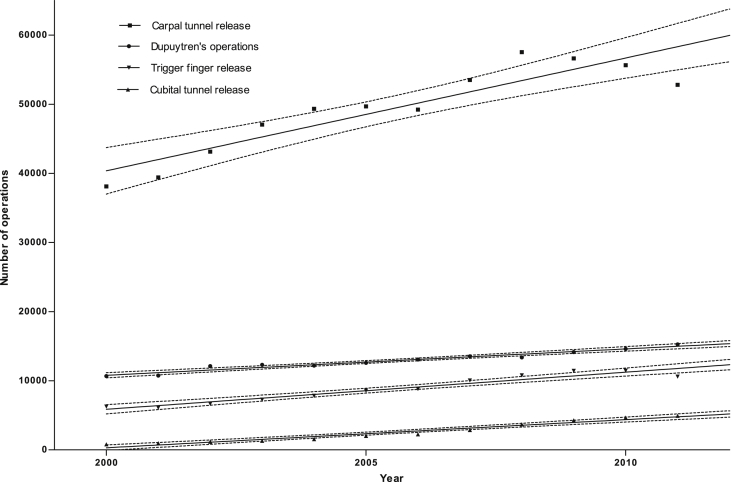
Absolute numbers of operations for four common hand conditions, 2000–2011. Solid lines represent the means, and dotted lines the 95% confidence intervals of linear regression analysis performed on the data. For carpal tunnel *r*^2^ = 0.83; Dupuytren's disease *r*^2^ = 0.96; trigger finger *r*^2^ = 0.93; cubital tunnel *r*^2^ = 0.95.

The number of operations performed for each hand disease between 2000 and 2011 reflects the increasing diagnoses, with carpal tunnel operations being the most common, followed by Dupuytren's disease operations, trigger finger release, and cubital tunnel release (Figure 7). Again, the greatest rate of growth is in carpal tunnel operations (slope mean 1632, 95% CI 1114–2150). The number of operations performed per diagnosis for 2000–2011 for each hand disease was calculated; the ratio for carpal tunnel, and trigger finger were constant at 0.97 and 1.10, respectively (Figure 4, *p* = 063 and *p* = 0.054 respectively). The ratio for cubital tunnel showed a linear increase from 0.31 to 0.67 over the study period (slope mean 0.033, 95% CI 0.031 to 0.035, *p* < 0.0001). This suggests that surgeons are increasingly likely to offer operative intervention rather than non-operative management for cubital tunnel syndrome, and accounts for some of the increase in surgery for this condition seen between 2000 and 2011.

A comparison of the slopes of pre-2008 versus post-2008 data for diagnoses and operations for each hand disease was calculated. The pre-2008 slopes were not significantly different to the post-2008 slopes for either diagnosis or operations for cubital tunnel release or trigger finger. Interestingly, for carpal tunnel release, the pre-2008 slope was significantly steeper than the post-2008 slope (*p* < 0.001), suggesting a trend towards decreased operative management.

### Predicted future operation rates

**Figure 8:**
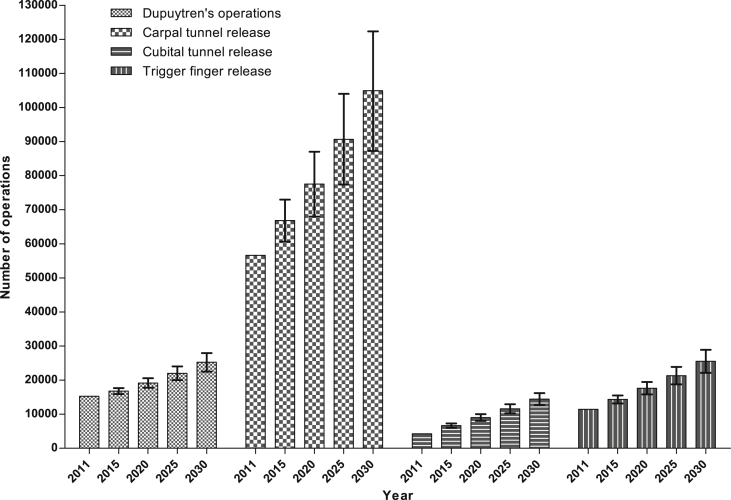
Predicted future operation rates for four common hand conditions. Error bars represent 95% confidence intervals.

Future operation rates for all hand diseases analysed are predicted to increase (Figure 8, Table 1). Most strikingly, for carpal tunnel operations in 2015 we calculate a 27% (95% CI 15–38) increase compared to 2011, and in 2030 we calculate the number of carpal tunnel release operations to have increased by 99% (95% CI 65–132) compared to 2011.

## Discussion

We analysed the change in demographics of four common hand conditions – Dupuytren's disease, carpal tunnel syndrome, cubital tunnel syndrome, and trigger finger – from 1998 to 2011, and used this data to predict the future workload for these conditions.

The most striking result from our analysis is the fact that all four conditions studied are increasing in both their absolute diagnosis numbers, the number of diagnoses per unit population, and the number of operations performed. Excepting cubital tunnel, we found that the increase in number of operations performed was not caused by an increase in the likelihood of having an operation once the diagnosis was made, as the ratio of operations to diagnoses remained constant. For cubital tunnel, we noted an increasing ratio of operations to diagnoses, suggesting surgeons are increasingly likely to provide operative intervention for cubital tunnel syndrome. The analysis of slopes of pre- and post-2008 diagnostic and operative data showed no statistical differences for cubital tunnel, or trigger finger. Interestingly, analysis of the regression slopes for both the diagnosis and operative management of carpal tunnel syndrome revealed that the post-2008 slope was significantly less steep than the pre-2008 data. This refutes the possibility that the introduction of the 18 week wait policy in the NHS in 2008 artificially increased the diagnosis and operation rate for this condition. For Dupuytren's disease, whilst the rate of diagnosis post-2008 seems to have increased, this has not translated to an increased operative management. One interpretation of this data is that hand surgeons are being referred more mild cases that do not warrant intervention.

Many predisposing factors are recognised for the conditions studied, and changes in prevalence of these factors will influence the results presented here. For example, obesity is a well-known risk factor for both carpal and cubital tunnel syndromes.^5,6^ The prevalence of obesity in England has increased from 13% to 24% in men, and 16–26% in women between 1993 and 2011.^7^ Continued increases will contribute to increased prevalence of carpal and cubital tunnel syndromes. Similarly, the prevalence of diabetes, a predisposing factor for carpal tunnel syndrome, Dupuytren's disease, and trigger finger^5,8,9^ was 2.6 million people in the UK in 2009, a figure which is expected to rise to 4 million by 2025.^10^ By contrast, the rate of smoking, which is known to contribute to the development of Dupuytren's disease, has decreased in prevalence since 1980, though it has remained stable between 2007 and 2010.^11^

The population of England is predicted to continue to slowly increase in the years to 2030, however the increase will not be uniform across all age groups, with older age groups constituting a larger proportion of the population over time. This is especially relevant to Dupuytren's disease, where the greatest diagnostic and operative workload, both currently and in the future, is in the 60–74 age group, compared to the 15–59 age group in the other conditions. The proportion of men and women in the population is predicted to stay the same, thus gender differences in prevalence of these diseases will not affect the results. Of the conditions studied, carpal tunnel syndrome provides the largest workload, and according to our calculations, this will remain so in the future. Carpal tunnel syndrome also has the fastest rate of increase of the four conditions studied.

The number of operations per diagnosis provides interesting data. The ratios of operations per diagnosis were stable for carpal tunnel syndrome, Dupuytren's disease and trigger finger. This gives us confidence that our linear regression will be accurate as there has been no change in trend for the operative treatment of any diagnosis. For trigger finger, the ratio was greater than one, which may be caused by the operation code ‘release of constricted tendon’ being applicable to more than one primary diagnosis, or that more than one digit is released for each diagnosis recorded. We believe this will not affect the validity of the results for trigger finger as the ratio has not changed over time, suggesting the proportion of operations for trigger finger has not changed. The ratio of diagnosis to operations for cubital tunnel syndrome showed a significant linear increase between 2000 and 2011, suggesting that there has been an increasing tendency to operate on cubital tunnel syndrome. It is likely that a plateau in this ratio will be reached at some stage, and therefore the increase in operations for cubital tunnel syndrome will be less than we have calculated. The timing and level of such a plateau are impossible to predict.

There are a number of potential confounders in our study. Firstly, concerns have been raised over the reliability of HES data, however it is the best data we have available, and indeed is used for policy planning by the UK government. Independent sector treatment centres are said to ‘over treat’ simple conditions, however this evidence is anecdotal thus would be impossible to account for in our analysis. The potential effect of the introduction of the 18 week wait has already been addressed and appears not to have artificially inflated the predicted operation rates.

Taken as a whole, our data are consistent with studies from the USA, that have demonstrated increasing demand for orthopaedics, neurosurgery, cardiothoracic surgery, general surgery, and surgical oncology.^12–15^ However, these previous data have simply taken account of the changing population demographics, assuming a constant disease prevalence. Our data integrate the population demographic changes with changing incidence of disease diagnosis and surgery over time to provide a more accurate estimate of future surgical workload.

Combining all four hand conditions studied, we have calculated that there will be almost a doubling in operative workload by 2030 compared to 2011, from 87,582 to 170,166 operations. This data adds weight to the argument that with the ageing population there will be a disproportionate increase in surgical procedures commonly required in elderly people.^3^ With such increases in demand there must be adequate service provision planning, integral to which is workforce planning. The recent trend towards supply-demand model for surgical training posts^4^ means that hand surgery needs to become a much larger sub-speciality. It is already recognised that surgeons tend to choose their speciality early compared to other specialities,^16^ thus hand surgery must work hard to attract students and junior doctors at an early stage of their training.

Service provision planning must also include the efficient use of the most cost effective treatments. For all of the conditions studied, more minimally invasive treatment modalities are becoming more widely used. For example, percutaneous needle fasciotomy and collagenase injection can be used to treat selected Dupuytren's Disease cases in an outpatient setting; trigger digits can be treated percutaneously; and carpal tunnel release is commonly performed under local anaesthesia in a minor procedure room setting.

In order to evaluate which treatments are most cost effective, high quality research evidence is required. However, there is a paucity of research funding allocation to musculoskeletal disorders – for example, in 2012/13 the UK Medical Research Council allocated just 2.5% of its research budget to musculoskeletal research.^17^ Furthermore, a recent analysis of global trends in hand and wrist research has shown that from 1988 to 2007 there has been no overall change in research volume. Encouragingly, there has been a relative increase in level I evidence, though it still remains well below the volume of level IV evidence production.^18^ The lack of research and adequate funding allocation to musculoskeletal conditions lead to the launch of the WHO Bone and Joint Decade 2000–2010 to try to increase awareness and funding for musculoskeletal conditions. We propose that there remains, beyond the Bone and Joint Decade, an urgent need to conduct high quality research, particularly randomised control trials to determine the best treatment options for common hand conditions. Integrated into these studies must be health economic analysis to determine which are the most cost effective. Combining such studies with adequate workforce planning will allow healthcare services to cope with the demands for hand surgery intervention from the ageing population of the future.

Finally, the methods presented here are more generally applicable to other sub-specialties. Similar analyses will help influence and inform workforce planning efforts in order to train adequate surgeons to cope with the demands of an ageing population.

## Ethical approval

Not required.

## Conflict of interest statement

No benefits in any form have been received or will be received from a commercial party related directly or indirectly to the subject of this article.

## Figures and Tables

**Table 1:** Calculated number of operations for each common hand condition 2015 to 2030. Figures shown represent the mean (95% confidence intervals).

Year	Dupuytren's operations	Carpal tunnel operations	Cubital tunnel release	Trigger finger release
2015	16797 (15893–17683)	66833 (60614–72948)	6664 (6005–7291)	14358 (13169–15510)
2020	19155 (17748–20563)	77506 (67998–87018)	9022 (8043–10002)	17618 (15803–19436)
2025	21998 (19995–24024)	90630 (77367–104020)	11575 (10247–12938)	21311 (18768–23896)
2030	25242 (22508–27929)	104922 (87236–122350)	14452 (12666–16167)	25550 (22107–28908)

## Appendix A Supplementary data

The following is the Supplementary Data Spreadsheet related to this article. This spreadsheet contains the data derived from Office for National Statistics and Hospital Episode Statistics data used in the calculations in this paper.
